# Integrative Multi-Omics Analysis Reveals Transcriptomic and Metabolic Remodeling Associated with Enhanced Peanut Nodulation Under Arbuscular Mycorrhizal Fungal Inoculation and Calcium Application

**DOI:** 10.3390/plants15172640

**Published:** 2026-08-28

**Authors:** Liyu Yang, Qi Wu, Haiyan Liang, Miao Liu, Pu Shen

**Affiliations:** Shandong Peanut Research Institute, Shandong Academy of Agricultural Sciences, P.R. China 126 Wannianquan Road, Qingdao 266100, China; liyuyang@whu.edu.cn (L.Y.); 15094073735@163.com (H.L.); shenpupeanut@126.com (P.S.)

**Keywords:** symbiotic efficiency, arbuscular mycorrhizal fungi, calcium regulation, transcriptional-metabolic remodeling, sustainable agriculture

## Abstract

Peanut (*Arachis hypogaea* L.) yield depends on biological nitrogen fixation, but the molecular mechanisms underlying the combined effects of arbuscular mycorrhizal fungi (AMF) and calcium fertilizer on nodulation remain unclear. Here, we used integrated transcriptomic and metabolomic analyses to investigate potential mechanisms in peanut roots. Compared with the non-inoculated control, AMF inoculation alone was associated with a 22.1% higher nodule number per plant. The combined application of AMF and CaO showed a 35.9% higher nodulation than AMF alone, and a 30.9% higher AMF colonization rate than AMF alone was also observed. Mechanistically, AMF colonization was associated with enhanced carbon-nitrogen metabolic profiles and up-regulation of phenylpropanoid metabolism-related pathways, suggesting a potential role in providing energy, carbon skeletons, and signaling molecules for nodule formation. Calcium fertilizer correlated with strengthening of the glyoxylate cycle and pentose phosphate pathway, possibly contributing to the energy supply for nodulation. It also affected genes related to protein secretion and lipid metabolism, with observed changes in membrane lipids and transport metabolites, which may enhance symbiotic interface function. This study reveals the multi-level mechanisms through which AMF and calcium fertilizer collectively promote peanut nodulation, providing a systems-level perspective on plant–microbe–nutrient relationships during symbiosis. Our findings offer new insights for sustainable agriculture by reducing chemical nitrogen inputs and promoting nodulation in legumes.

## 1. Introduction

Peanut, a globally important cash crop with annual production exceeding 51 million tons, plays a vital role in global oil and protein supplies [1]. While expanding cultivation areas have increased economic returns, this expansion has also led to an alarming rise in nitrogen fertilizer application, which has simultaneously raised production costs and caused environmental pollution through nitrate leaching and greenhouse gas emissions. As a legume crop, peanut has the unique ability to form symbiotic relationships with nitrogen-fixing rhizobacteria, an ability that can reduce dependence on chemical nitrogen fertilizers [2]. However, peanut production is frequently constrained by soil nutrient deficiencies and environmental stresses, particularly calcium limitation and soil acidification, which not only impair root development but also contribute to substantial yield losses worldwide [3,4]. Therefore, improving the symbiotic relationship and promoting nodulation in peanut have become key to reducing the nitrogen fertilizer dependence during peanut cultivation and protecting the soil ecosystem.

AMF, ubiquitous soil microorganisms, form mutualistic symbiotic associations with most terrestrial plants through arbuscular mycorrhizae. The relationship between legume root systems and AMF represents one of the most ancient and ecologically significant plant–microbe relationships [5]. Recent studies demonstrate that legume nitrogen fixation involves complex tripartite relationships among host plants, rhizobia, and AMF [6,7]. AMF can promote legume nodulation and enhance the efficiency of symbiotic nitrogen fixation in legumes through multiple mechanisms [8]. Specifically, AMF improves phosphorus uptake and facilitates direct transport of Pi to the root cortex via mycelium, thereby avoiding inhibition of rhizobial infestation by high phosphorus in the rhizosphere [9]. In addition, AMF up-regulates sucrose transporter proteins, increasing carbon allocation to roots and supplying essential metabolites to support rhizobial nitrogen fixation [10,11]. Moreover, AMF triggers the secretion of strigolactones from plant roots, which in turn promotes rhizobial chemotaxis and enhances bacterial activity in the nodule meristematic zone, ultimately facilitating nodule formation [12,13]. Previous studies have shown that the combined action of AMF and rhizobia contributes to peanut nodulation [14]. Inoculation with AMF resulted in enhanced nodulation in peanut [15]. Therefore, increasing AMF inoculation rates in plants is important for promoting nodulation, which varies widely due to environmental factors and agricultural practices. This variation highlights the need for effective measures that can realize the benefits of AMF inoculation in actual growing practices.

The efficiency of nodulation and nitrogen fixation in legume crops can be affected by various environmental factors, one of which is soil nutrient content. Calcium (Ca) is an essential macronutrient that plays a key role in many plant physiological processes, including cell wall stabilization [16], membrane integrity maintenance [17] and signal transduction [18]. In legume symbioses, Ca^2+^ serves as a crucial signaling role in nodule initiation and development. Among the early recognition events of rhizobacteria and plant roots, the Ca^2+^ signaling system is triggered by mutualistic symbiosis [19]. Nuclear-localized calcium- and calmodulin-dependent protein kinases (CCaMKs) decode nuclear-associated calcium oscillations, which phosphorylate downstream targets like CYCLOPS to initiate nodule organogenesis [20]. At the nodule formation stage, Ca^2+^ is essential for the growth of infection threads and for the differentiation of bacteroids [21]. In addition, research on various legume species has demonstrated that the efficiency of biological nitrogen fixation is directly modulated by calcium levels within plant tissues. Insufficient calcium concentrations reduced nitrogenase activity in mature nodules of Vicia faba and soybean [22,23,24]. In addition to playing a crucial role in optimizing nitrogen fixation in legume nodules, calcium also plays an active and critical role in promoting AMF invasion of the plant root. The expression of arbuscular mycorrhiza-specific marker genes (such as *AhCaM*, *AhCDPK*, *AhRAM1*, and *AhRAM2*), which was suppressed under Ca^2+^ deprivation, was restored upon exogenous Ca^2+^ application [25]. Despite the recognized significance of Ca, the combined effects of exogenous Ca application on peanut nodulation, particularly when combined with AMF, involve complex regulatory networks that encompass gene expression and metabolic and plant phenotypic adaptations, and remain poorly understood.

In this study, it was hypothesized that the application of exogenous calcium could further promote nodulation and symbiotic performance in peanut root systems through AMF inoculation. This hypothesis is particularly relevant under stress conditions such as calcium-deficient or acid soils, where nodulation performance is frequently constrained, which ultimately leads to considerable losses in peanut pod yield and quality. To address this, we evaluated the combined application of exogenous calcium and AMF inoculation on peanut root nodulation, and specifically, investigated the underlying mechanisms through comparative analyses of gene expression and metabolic profiles under different treatments, with a particular focus on Ca-AMF crosstalk in nodulation-related pathways. By elucidating these complex relationships, this study aims to support the development of sustainable agricultural practices that optimize nutrient utilization and improve crop productivity.

## 2. Results

### 2.1. AMF Colonization

To evaluate the establishment of AMF symbiosis, root colonization rates were assessed for the three experimental treatments: (i) non-inoculated control without CaO (Control), (ii) AMF inoculation alone (AMF), and (iii) AMF inoculation combined with CaO application (AMF+CaO). No AMF colonization was observed in the non-inoculated Control plants. The AMF-treated plants showed an average colonization rate of 35.62 ± 1.27%, while the AMF+CaO treatment showed a colonization rate of 46.61 ± 1.50% (Table S1), with a numerical difference of 30.9% between the two group means. However, the biological interpretation of this difference warrants further investigation, as the CaO-only treatment was not included in this study.

### 2.2. Phenotypic Changes in Peanut Roots Under AMF Inoculation and Calcium Fertilizer Application

**Figure 1:**
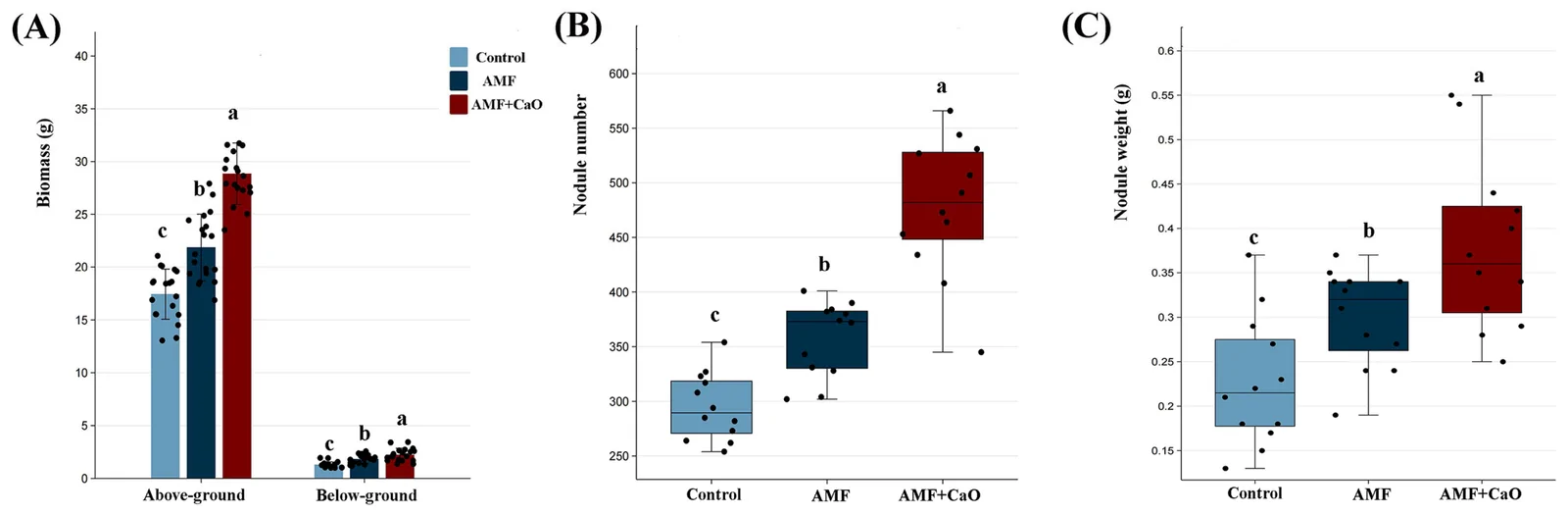
Effects of different treatments on peanut biomass, nodule number, and nodule weight. (**A**) Shoot and root dry weight (g·plant^−1^). (**B**) Nodule number (nodules·plant^−1^). (**C**) Nodule weight (g·plant^−1^). Control, non-inoculated; AMF, AMF inoculation; AMF+CaO, AMF + calcium oxide. Data are presented as mean ± SD. For (**A**), *n* = 19 independent pots per treatment (one plant per pot); for (**B**,**C**), *n* = 12 independent pots per treatment (one plant per pot). In all cases, each pot contributed one plant, and each data point represents an independent pot. Statistical significance was determined by one-way ANOVA followed by Tukey’ s post hoc test. Different letters indicate significant differences among treatments at *p* < 0.05.

Compared with normally growing peanuts (Control), single inoculation with AMF (AMF) significantly increased root nodulation levels (Figure 1A,B). Roots of AMF exhibited 354 ± 33 nodules·plant^−1^, representing a 22.1% increase (*p* = 0.007) over Control (290 ± 28 nodules·plant^−1^). Dry weight increased from 0.22 ± 0.07 g·plant^−1^ to 0.30 ± 0.06 g·plant^−1^, a 36.4% increase (*p* = 0.012). When calcium fertilizer was applied in conjunction with mycorrhizal symbiosis (AMF+CaO), the nodule number reached 481 ± 61 nodules·plant^−1^, which was 65.9% and 35.9% greater than the Control and AMF treatments, respectively (*p* < 0.001). Additionally, the dry weight of root nodules reached 0.38 ± 0.09 g·plant^−1^, which represented increases of 72.7% and 26.7% over the Control and AMF, respectively (*p* = 0.003). Significant differences were observed among all three treatments.

### 2.3. Gene Expression Profiles of Peanut Roots Under Different Treatments

**Figure 2:**
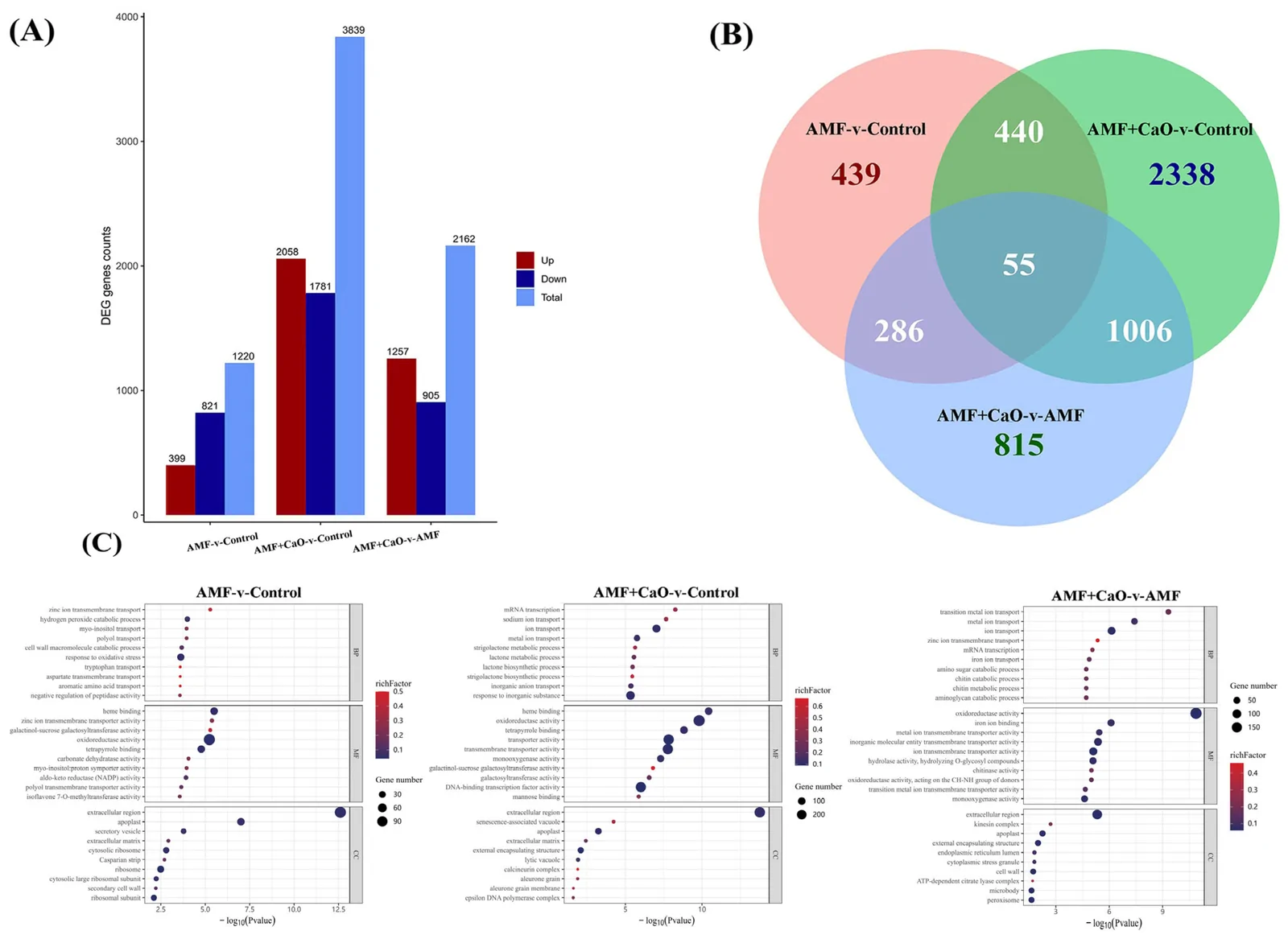
Transcriptomic profiling and GO enrichment analysis under different treatments. (**A**) Number of differentially expressed genes (DEGs) in AMF-v-Control and AMF+CaO-v-Control comparisons. Red, light blue, and dark blue dots denote upregulated, downregulated, and total DEGs, respectively. (**B**) Venn diagram showing the overlap of differentially expressed genes among the three comparisons. Pink, green, and blue circles represent DEGs for AMF vs. Control, AMF+CaO vs. Control, and AMF+CaO vs. AMF comparisons, respectively. (**C**) GO enrichment analysis for AMF-v-Control, AMF+CaO-v-Control, and AMF+CaO-v-AMF comparisons. The size of dots represents gene number, and the color gradient indicates the rich factor.

To elucidate the transcriptional response of peanut roots in response to arbuscular mycorrhizal fungus (AMF) colonization and its combined application with CaO, we performed a comparative transcriptomic analysis across three treatment groups: Control, AMF, and AMF+CaO. To assess the overall structure of the transcriptomic datasets and evaluate the reproducibility among biological replicates, principal component analysis (PCA) was conducted on the normalized expression profiles of all samples. As shown in Figure S1, the PCA score plot revealed a clear and distinct separation of the three treatment groups along the first two principal components, which collectively accounted for 88.83% of the total variance (PC1: 73.88%, PC2: 14.95%). Biological replicates within each treatment formed tight, cohesive clusters, demonstrating high intra-group reproducibility and confirming the reliability of the RNA-seq dataset for downstream differential expression and functional enrichment analyses. In this study, we identified a total of 56,739 genes, among which 5379 genes exhibited differential expression across different comparisons (AMF-v-Control, AMF+CaO-v-Control, AMF+CaO-v-AMF) (Figure 2A; Table S2). The transcriptomic data were further validated by qRT-PCR (Figure S2). Except for 55 genes that showed differential expression in all comparisons, the vast majority of differentially expressed genes (DEGs) were unique to specific comparisons. As shown in Figure 2B, the AMF+CaO-v-Control group exhibited the highest number of differentially expressed genes across all comparisons. The number of DEGs in the AMF+CaO-v-AMF group was nearly double that of the AMF-v-Control group. The combined application of calcium fertilizer and AMF inoculation led to more pronounced changes in gene expression within peanut root systems compared with AMF inoculation alone. To systematically dissect the biological functions underlying transcriptomic alterations induced by calcium application and AMF inoculation, we performed Gene Ontology (GO) enrichment analysis covering three categories: biological process (BP), molecular function (MF), and cellular component (CC), across three comparison groups (AMF-v-Control, AMF+CaO-v-Control, AMF+CaO-v-AMF) (Figure 2C). For AMF-v-Control, DEGs were significantly enriched in biological processes including zinc ion transport, oxidative stress response and cell wall catabolism; dominant molecular functions included oxidoreductase and ion transporter activities, while enriched cellular components were extracellular region, apoplast and ribosome. The AMF+CaO-v-Control group showed stronger enrichment signals, with unique enriched pathways of strigolactone biosynthesis and transcriptional regulation besides diverse ion transport processes. Its top molecular functions involved transcription factor and transmembrane transporter activities, and senescence-associated vacuole was the most prominent cellular component. The comparison of AMF+CaO-v-AMF revealed that CaO supplementation under AMF colonization specifically enriched pathways related to transition metal ion transport and chitin catabolism. Accordingly, chitinase and metal transporter activities were enriched in the molecular function (MF) category, while endoplasmic reticulum and peroxisome were enriched in the cellular component (CC) category. These results suggest that CaO addition under AMF symbiosis is associated with the regulation of ion homeostasis, as well as hormone biosynthesis and symbiosis-related metabolic pathways.

**Figure 3:**
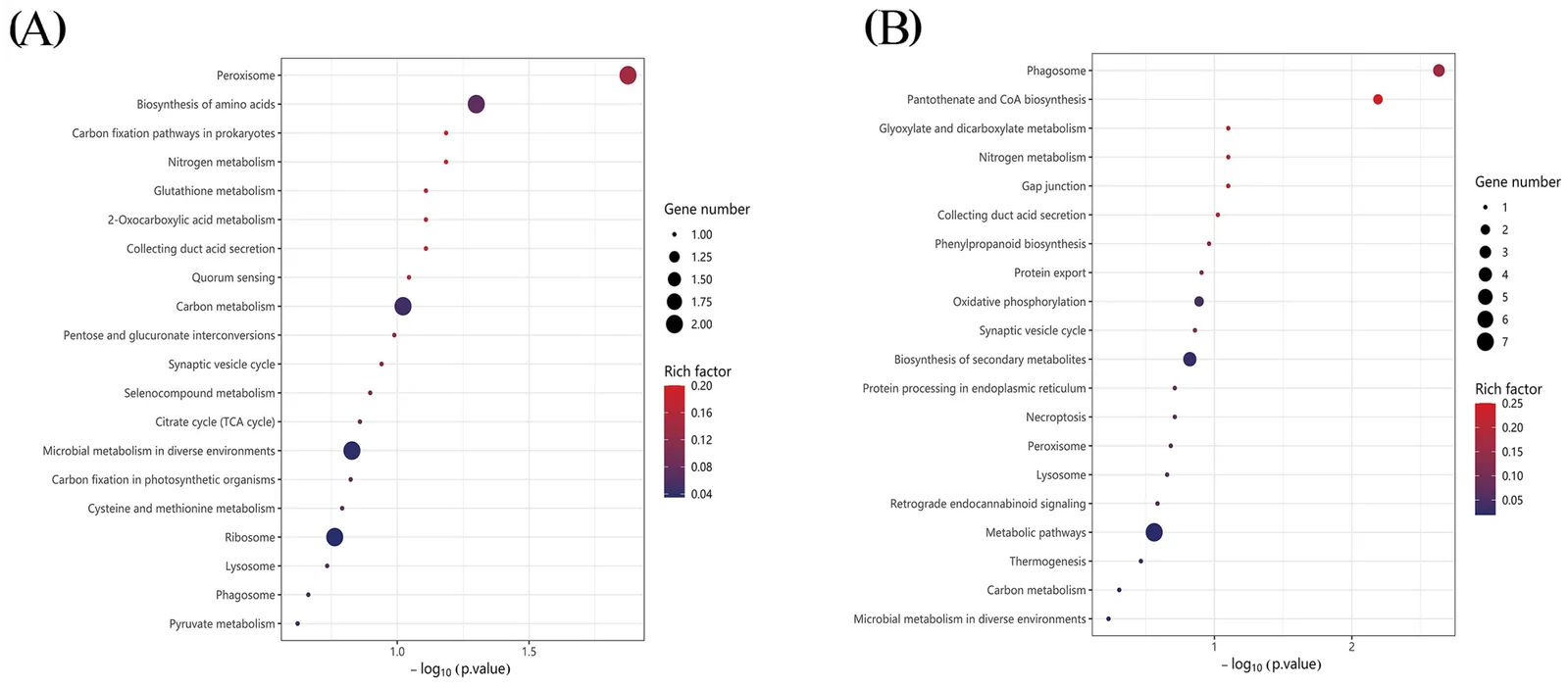
KEGG pathway enrichment analysis for AMF-v-Control (**A**) and AMF+CaO-v-Control (**B**). The size of dots represents gene number, and the color gradient indicates the rich factor. The redder the color, the higher the concentration.

Through enrichment analysis of gene functions related to the stress response to arbuscular mycorrhizae inoculation, we found that DEGs responding to inoculation were primarily enriched in carbon metabolism, pyruvate metabolism, and nitrogen metabolism pathways (Figure 3; Table S3). Under AMF inoculation combined with calcium fertilizer application, DEGs predominantly clustered in carbon metabolism, protein export, glyoxylate and dicarboxylate metabolism, and nitrogen metabolism (Figure 3; Table S3). The protein export pathway was uniquely enriched in the AMF+CaO treatment, while glyoxylate and dicarboxylate metabolism also appeared specifically under this combined condition.

### 2.4. Changes in Metabolic Characteristics of Peanut Root Systems Under Different Treatment Conditions

**Figure 4:**
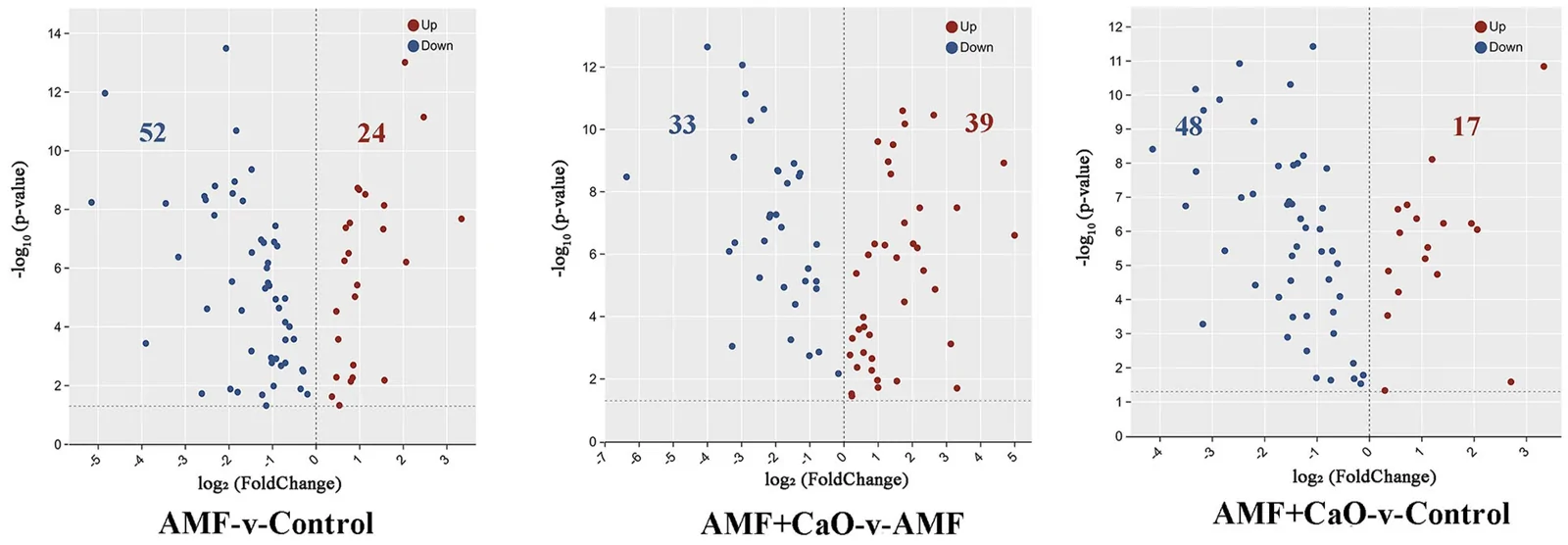
Volcano plots displaying differentially accumulated metabolites in AMF-v-Control, AMF+CaO-v-Control, and AMF+CaO-v-AMF comparisons. Red and blue dots represent significantly up-regulated and down-regulated metabolites, respectively.

Metabolites are direct participants in plant physiological and biochemical reactions. Changes in the metabolome under different cultivation treatments largely reflect plant responses to various treatments. Principal component analysis (PCA) of the peanut root metabolomics data revealed that samples from the same treatment clustered together, while those from different treatments were distinctly separated (Figure S3). These results suggest that AMF symbiosis significantly remodels the host root metabolic network, and that calcium application exerts an additional modulatory effect on this mycorrhiza-induced metabolic reprogramming. A total of 1164 metabolites were identified in this study. Among them, 76 metabolites (24 up-regulated, 52 down-regulated) in AMF-v-Control, 65 metabolites (17 up-regulated, 48 down-regulated) in AMF+CaO-v-Control, and 72 metabolites (39 up-regulated, 33 down-regulated) in AMF+CaO-v-AMF conditions exhibited differential accumulation (defined by VIP score > 1 and *p*-value < 0.05) (Figure 4; Table S4).

**Figure 5:**
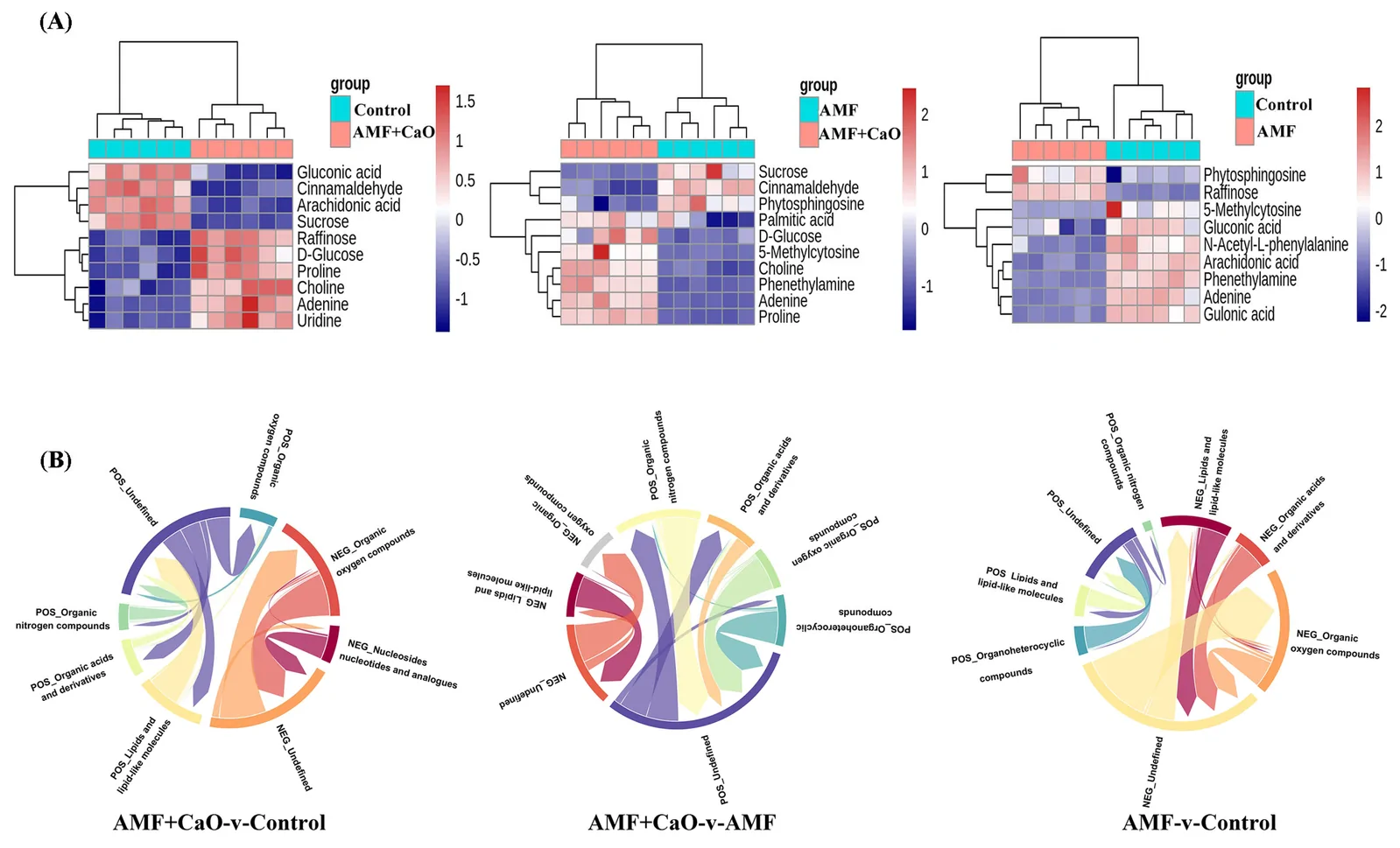
Heatmap and Chord overview of differential metabolites across AMF-v-Control, AMF+CaO-v-Control, and AMF+CaO-v-AMF. (**A**) Clustering heatmap of the top 10 differential metabolites in AMF-v-Control, AMF+CaO-v-Control, and AMF+CaO-v-AMF comparisons. The y-axis shows metabolites with significant differences in expression, and the x-axis shows sample information. The colored blocks at different positions represent the relative expression levels of the corresponding metabolites; red indicates relatively high expression, while blue indicates relatively low expression. Metabolites with similar expression patterns are grouped together in the same cluster on the left. (**B**) Chord diagram of metabolite class co-regulation based on significant correlations (|r| > 0.8, *p* < 0.05). Chord width is proportional to the number of significantly correlated metabolite pairs connecting two classes (transition weight); wider chords indicate stronger inter-class co-regulation. Weight matrices are provided in Table S5.

Hierarchical clustering heatmaps displayed the relative abundance of differential metabolites across AMF-v-Control, AMF+CaO-v-Control and AMF+CaO-v-AMF groups. Biological replicates of each treatment clustered tightly, demonstrating high intra-group consistency and clear metabolic divergence between treatments. For AMF-v-Control, AMF inoculation treatment elevated arachidonic acid and phenylalanine derivatives, while Control maintained higher phytosphingosine and raffinose. In AMF+CaO-v-Control, the combined calcium and mycorrhizae treatment drastically accumulated gluconic acid, cinnamaldehyde, and arachidonic acid, whereas D-glucose, adenine and uridine were enriched in Control. The AMF+CaO-v-AMF comparison identified metabolites specifically induced by mycorrhizal inoculation, including sucrose, phytosphingosine, and 5-methylcytosine, while proline and palmitic acid were more abundant in AMF samples (Figure 5). Based on the chord diagrams generated for the three comparative groups, the metabolic class compositions revealed distinct biochemical signatures that reflect underlying biological process differences. In the AMF-v-Control comparison, the negative ion mode was predominantly characterized by lipids and lipid-like molecules, organic oxygen compounds, and organic acids and derivatives, suggesting that carbohydrate metabolism and lipid homeostasis are major contributors to the metabolic landscape. The presence of organic acids points to active tricarboxylic acid cycle flux, while lipids indicate involvement in membrane structure and energy storage. In positive mode, organoheterocyclic compounds and organic nitrogen compounds emerge prominently. In the AMF+CaO-v-Control comparison, nucleosides, nucleotides, and analogues appear alongside organic oxygen compounds, highlighting enhanced nucleic acid metabolism and energy transfer processes. Lipids, organic acids, and organic nitrogen compounds were strongly represented, indicating coordinated involvement of lipid biosynthesis, and amino acid metabolism. The AMF+CaO-v-AMF comparison shows a more balanced distribution across multiple classes. Organic oxygen compounds, organic nitrogen compounds, organic acids, and organoheterocyclic compounds form a tightly represented group, reflecting integrated regulation of amino acid metabolism, organic acid cycling, and heterocyclic compound turnover.

**Figure 6:**
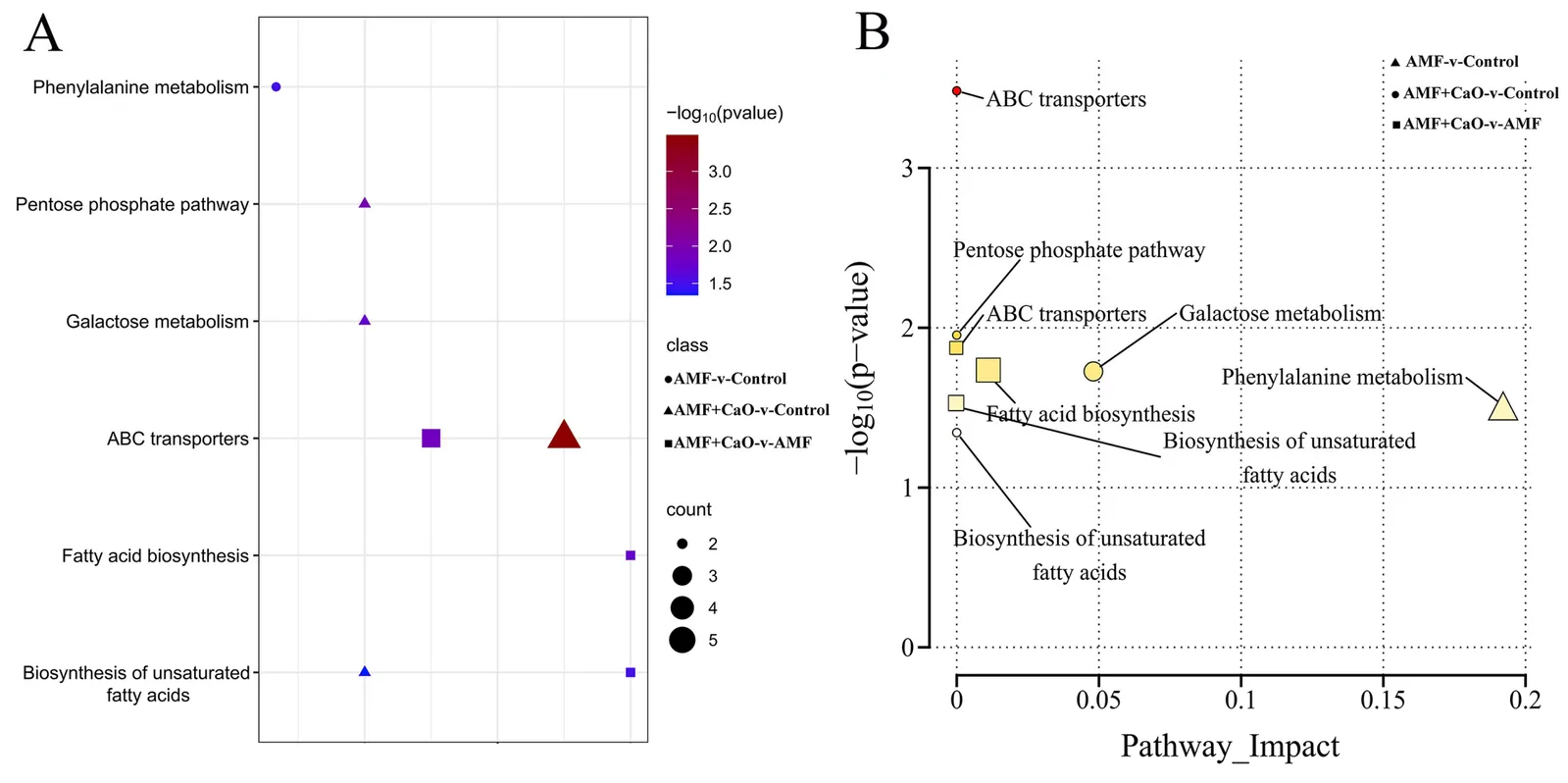
KEGG pathway enrichment analysis of differentially expressed metabolites across different comparisons. (**A**) Bubble chart of KEGG enrichment pathway. The different shapes represent three sets of comparison pairs; the color of each block corresponds to the significance of enrichment, with redder shades indicating higher significance of pathway enrichment; the size of each shape represents the number of differentially expressed metabolites within the pathway. (**B**) The pathway impact dot plot of KEGG enrichment analysis. The x-axis represents pathway significance, and the y-axis represents enrichment significance; the higher the value, the more significant the pathway. The shape of the graph distinguishes between the three comparison groups (circle: AMF-v-Control; triangle: AMF+CaO-v-Control; square: AMF+CaO-v-AMF).

Metabolomics has found extensive application in plant science, providing a more intuitive understanding of plant stress response mechanisms by amplifying subtle changes in gene and protein expression at the metabolic level. Analysis of differential metabolites produced in peanut roots under different experimental treatments revealed that, compared to Control, metabolic differences induced by arbuscular mycorrhizae inoculation were primarily associated with the phenylalanine metabolism pathway (Figure 6A). In contrast, differential metabolites resulting from arbuscular mycorrhizae inoculation combined with calcium fertilizer application were assigned to the pentose phosphate pathway, galactose metabolism, ABC transporters, and the biosynthesis of unsaturated fatty acids. The pathway impact dot plot (Figure 6B) reveals that the phenylalanine metabolism pathway from the AMF-v-Control group exhibits the largest pathway impact value and the largest marker size, signifying that this pathway is the primary driver of metabolic shifts under sole AMF inoculation. In the AMF+CaO-v-AMF group, ABC transporters are positioned at the highest position on the Y-axis, and their marker also reflects a substantial functional impact. The pentose phosphate pathway likewise shows strong statistical significance, whereas galactose metabolism has a moderately high pathway impact value paired with intermediate enrichment significance. Meanwhile, fatty acid biosynthesis and the biosynthesis of unsaturated fatty acids are also significantly enriched pathways in this combined treatment group, collectively confirming that the co-application of arbuscular mycorrhizae and calcium reshapes metabolic profiles in peanut roots through the regulation of carbon metabolism, transmembrane substance transport, and lipid synthesis.

**Figure 7:**
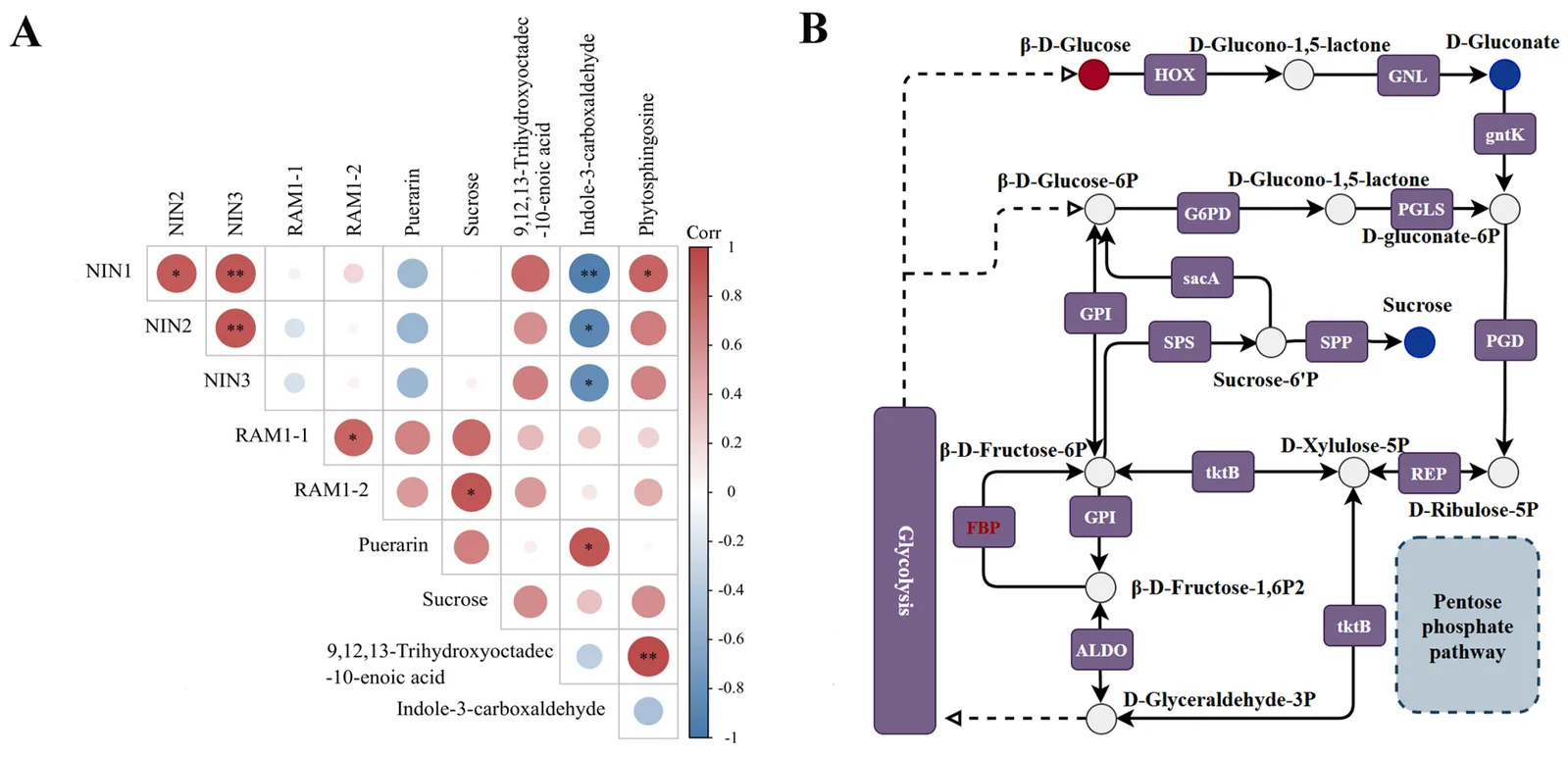
Integrated transcriptome–metabolome correlation analysis. (**A**) Pearson correlation heatmap of symbiotic signaling-related DEGs (three *NIN* genes and two *RAM1* genes) and key differential metabolites. Only the upper triangle of the matrix is shown for clarity. Red and blue colors indicate positive and negative correlations, respectively. The intensity of color and size of circles represent the correlation coefficient. * *p* < 0.05, ** *p* < 0.01. *NIN1*: *AH13G06340*; *NIN2*: *AH03G04320*; *NIN3*: *AH17G00510*; *RAM1-1*: *AH16G30060*; *RAM1-2*: *AH06G24360*. (**B**) The pentose phosphate pathway in AMF+CaO-v-Control comparison. Circles represent metabolites. Red and blue circles denote significantly up-regulated and down-regulated metabolites, respectively. Solid arrows indicate direct metabolic steps and products, while dotted arrows represent indirect or multi-step relationships. Purple-gray squares represent genes, and red text indicates significantly up-regulated genes. FBP: fructose-1,6-bisphosphatase I (*AH20G09090*); GPI: glucose-6-phosphate isomerase; GNL: Gluconolactonase; ALDO: fructose-bisphosphate aldolase; HOX: hexose oxidase; PGD: 6-phosphogluconate dehydrogenase; PGLS: 6-phosphogluconolactonase; G6PD: glucose-6-phosphate 1-dehydrogenase; gntK: Gluconokinase; TKT: Transketolase. SPP: Sucrose-6-phosphatase; SPS: Sucrose-phosphate synthase; sacA: beta-fructofuranosidase.

Given that NIN and RAM1 are key transcriptional regulators of symbiotic programs [26,27], we examined whether their expression changes were associated with specific metabolic shifts. Among the DEGs, we identified three *NIN* genes (*NIN1*–*NIN3*) and two *RAM1* genes (*RAM1-1* and *RAM1-2*) and also selected five representative DAMs covering distinct functional categories relevant to symbiosis: isoflavonoid signaling (Puerarin), soluble sugar/energy supply (Sucrose), fatty acid-derived signaling (9,12,13-trihydroxyoctadec-10-enoic acid), auxin-related regulation (Indole-3-carboxaldehyde), and sphingolipid signaling (Phytosphingosine). The three NIN genes exhibited strong positive correlations with 9,12,13-trihydroxyoctadec-10-enoic acid (r = 0.60–0.80) and Phytosphingosine (r = 0.67–0.83), but strong negative correlations with Indole-3-carboxaldehyde (r = −0.83 to −0.93). The two RAM1 genes displayed positive correlations with all these five DAMs. There was a strong positive correlations with Sucrose (r = 0.79–0.89) (Figure 7A; Table S6).

To identify potential convergence points between transcriptional and metabolic regulation, we cross-compared KEGG enrichment results from both omics datasets (Figure 7B; Table S6). The pentose phosphate pathway (PPP) was the only pathway concurrently enriched with both a DEG and DAMs. We therefore examined the correlation patterns of *AH20G09090* (encoding fructose-1,6-bisphosphatase I, FBP) with PPP-related metabolites under different treatments. In the AMF-v-Control comparison, *AH20G09090* showed a significant positive correlation with gluconic acid (r = 0.8156, *p* = 0.0478) and a negative correlation with sucrose that approached statistical significance (r = −0.7651, *p* = 0.0761), while its correlation with D-glucose was weak and non-significant (r = −0.4256, *p* = 0.4008). In the AMF+CaO-v-Control comparison, however, this pattern shifted. *AH20G09090* showed a positive correlation with D-glucose accumulation that approached statistical significance (r = 0.8009, *p* = 0.0556), while its correlations with gluconic acid (r = −0.7499, *p* = 0.0861) and sucrose (r = −0.5796, *p* = 0.2284) were not statistically significant. A significant negative correlation was observed between D-glucose and gluconic acid (r = −0.8496, *p* = 0.0323), and a positive correlation between gluconic acid and sucrose was also detected (r = 0.7899, *p* = 0.0617, marginally non-significant but suggestive of a trend).

## 3. Discussion

The symbiotic nitrogen fixation between leguminous crops and rhizobia represents a crucial biological nitrogen fixation pathway in agricultural ecosystems, with its efficiency dependent on nodule formation and functional maintenance [28]. AMF, as widely distributed plant root symbionts, enhance plant phosphorus and nitrogen uptake while providing carbon sources to rhizobia by regulating plant carbon allocation [29]. Furthermore, calcium, as a central second messenger in plant cell signaling, is indispensable in nodulation factor-induced calcium oscillation pathways and cell wall construction at symbiotic interfaces, while also regulating carbon and nitrogen metabolism (such as pyruvate dehydrogenase activity and amino acid transport) [22,30]. Based on our integrated transcriptomic and metabolomic analyses, we observed potential molecular and metabolic signatures associated with the promoting effect of combined AMF and calcium fertilizer application on peanut nodulation. Notably, the concurrent enrichment of unsaturated fatty acid biosynthesis and ABC transporter pathways suggests a crucial role for AMF-mediated lipid transfer, providing candidate targets for future functional studies aimed at optimizing symbiotic performance.

The enrichment of unsaturated fatty acid biosynthesis and ABC transporters is particularly noteworthy in the context of AMF symbiosis. In *Medicago truncatula*, the establishment of functional arbuscules requires host plants to supply lipids to the fungus, a process dependent on the half-ABC transporters STR/STR2 for lipid efflux across the periarbuscular membrane, and on RAM2/FatM for the biosynthesis of fungal-accessible lipid species [31,32]. Our observation that these pathways are co-activated in peanut roots under AMF+CaO treatment strongly suggests that calcium fertilization may potentiate the lipid transfer from roots to intraradical hyphae, thereby enhancing AMF colonization efficiency and, consequently, the carbon/nutrient supply to rhizobia within nodules. Additionally, the up-regulation of full-size ABCG transporters, as reported in *Medicago* for strigolactone secretion [13], implies that calcium signaling might also amplify presymbiotic signal output, further coordinating the dual symbiosis. These possibilities merit targeted functional validation in future studies.

Nodules are specialized organs formed through the symbiotic relationship between legumes and rhizobia, serving as a prerequisite for symbiotic function. The nodulation process is a highly energy-consuming biological event involving multiple steps such as signal exchange, cellular remodeling, organogenesis, and functional maintenance, each requiring substantial ATP and metabolic support [33]. In the present study, peanut root systems exhibited significant transcriptional regulation and metabolic reorganization following AMF inoculation. Transcriptome analysis revealed that DEGs were significantly enriched in carbon metabolism and pyruvate metabolism pathways. AMF inoculation activated metabolic pathways related to ATP energy and carbon skeleton formation in peanut roots, thereby providing essential material support for subsequent cell division of nodule primordium formation and symbiotic signal transduction. The activation of carbon metabolism pathways may provide essential energy and carbon skeletons for establishing the symbiotic relationship between peanuts and AMF. As a key component of glycolysis, the enrichment of pyruvate metabolism may support material synthesis and energy supply during symbiosis by regulating respiration. The significant enrichment of nitrogen metabolism pathways further suggests that AMF inoculation may enhance nitrogen uptake and assimilation in peanut roots, which is crucial for nodule formation and symbiotic function. Previous studies have revealed that the phenylpropanoid metabolism pathway is significantly enriched in AMF-inoculated soybean roots, with the key gene *GmPAL2.4* encoding the initial and rate-limiting enzyme in this pathway, showing substantial up-regulation. Overexpression of *GmPAL2.4* markedly increased the expression of genes involved in the biosynthesis of these flavonoids and phenolic acids, thereby enhancing nodulation and symbiotic function in soybean [34]. The enrichment of phenylalanine metabolism serves as a crucial bridge linking AMF colonization and rhizobial recruitment. Phenylalanine serves as a direct precursor for flavonoid synthesis, and increased flavonoid accumulation, acting as potent inducers of rhizobial nodulation genes-signals stronger symbiotic recruitment cues released by roots into the rhizosphere [35]. Therefore, we hypothesize that AMF inoculation of peanut roots paves the way for subsequent symbiotic nodulation by optimizing the host’s energy-nutrient status and actively shaping the rhizosphere chemical signaling environment to favor rhizobial recognition.

Compared to AMF inoculation alone, the addition of calcium fertilizer to AMF inoculation induced more profound and specific systemic responses, revealing calcium’s central regulatory role in AMF-mediated symbiosis. The glyoxylate cycle converts lipids into carbohydrates, while dicarboxylic acids (including malic acid and succinic acid) serve as the preferred carbon sources for rhizobial cells [36]. In the present study, glyoxylate and dicarboxylate metabolism was activated following AMF inoculation and calcium fertilizer application, suggesting calcium fertilizer treatment may optimize mobilization of carbon storage compounds and directly enhance energy supply for symbiotic function. Furthermore, DEGs were significantly enriched in protein export pathways, strongly suggesting that secretion activities related to symbiotic membrane formation, nutrient exchange, or defense regulation were specifically enhanced. This facilitates the establishment and maintenance of a more effective symbiotic interface [37]. The activation of protein export pathways suggests that calcium signaling may regulate the secretion of signaling molecules or functional proteins from root cells to symbionts, enhancing mutual signal exchange and material transport. The enrichment of glyoxylate and dicarboxylate metabolism, which are critical branches of carbon metabolism, may provide the symbiotic system with more diverse carbon utilization pathways, optimizing carbon flux allocation efficiency. The enrichment of DAMs in the pentose phosphate pathway ensured the supply of NADPH and nucleotide precursors, favoring rapid proliferation of nodule cells. Chinnasamy et al. [38] found that increased unsaturated fatty acids in pea nodules enhance membrane fluidity, while ABC transporters primarily mediate transmembrane transport of lipids, signaling molecules, and nutrients, playing a crucial role in beneficial symbiosis [39,40]. In alfalfa (*Medicago sativa* L.), MtABCG56 is a cytokinin efflux transporter localized to the plasma membrane, expressed in roots and nodules, and plays a crucial role in nodule formation [41]. In the AMF+CaO-v-AMF comparison, DAMs showed significant enrichment in biosynthesis of unsaturated fatty acids and ABC transporters, collectively indicating major restructuring of cell membrane structure and function. This membrane system upgrade likely substantially enhances material exchange efficiency and signal transmission rates between symbiotic partners. The concurrent up-regulation of ABC transporters and lipid metabolism upon calcium fertilizer is consistent with calcium’s role in modulating membrane-associated processes in plants. Enhanced transporter activity may contribute to the secretion of symbiotic signals such as flavonoids and strigolactones, while also supporting lipid exchange at the plant-fungus interface. Together with the parallel activation of phenylpropanoid and pentose phosphate pathways, these changes provide both signaling molecules and metabolic precursors that favor the establishment of an effective symbiotic association. Therefore, we hypothesize that calcium ions introduced by calcium fertilizer application, acting as a crucial second messenger, may regulate the expression or activity of ABC transporter family genes. This accelerates the transmembrane transport of key substances like lipids and nitrogenous compounds, providing rhizobial cells with more abundant carbon sources and energy supply. The sustained significant enrichment of both carbon and nitrogen metabolism pathways indicates that calcium fertilizer may further enhance peanut root energy production and nitrogen uptake assimilation capacity, potentially contributing to symbiotic efficiency and providing a more robust material and energy foundation for peanut growth and development. Together, these results suggest that calcium addition to AMF inoculation enhances symbiotic efficiency through a multi-level regulatory mechanism that optimizes the coordinated operation of carbon-nitrogen metabolism, consolidates the symbiotic advantage under activated conditions, and ultimately establishes a robust physiological foundation for effective symbiotic function and growth development of peanut plants.

The association between legumes and rhizobia, as well as arbuscular mycorrhizal fungi (AMF), is crucial for nodulation and symbiotic function of leguminous crops and relies heavily on successful AMF colonization [42,43]. In this study, compared with plants not inoculated with AMF, inoculation with AMF alone increased nodulation in peanuts by 22.1%, which is consistent with previous findings. Calcium, as a central second messenger in plant cell signal transduction, is indispensable in nodulation factor-induced calcium oscillation pathways and the formation of the symbiotic interface cell wall [21,44]. A recent study on calcium-mediated nodulation regulation have demonstrated that calcium application alone can substantially enhance peanut nodulation, with increases of up to 84.0% [45]. Our experimental soil presented a different challenge, with sub-deficient calcium (238 mg·kg^−1^) but excessively high phosphorus (61.06 mg·kg^−1^), a condition known to suppress AMF colonization and function. Under this high-phosphorus background, AMF colonization alone reached only 35.62%, yet AMF inoculation still achieved a 22.1% increase in nodulation. More importantly, the addition of CaO to AMF inoculation not only increased colonization rate by 30.9% (from 35.62% to 46.61%) but also further boosted nodulation by 35.9% compared with AMF alone. These results suggest that while calcium alone is effective in calcium-deficient soils, the AMF+CaO combination offers a distinct advantage in soils where multiple nutrient constraints coexist, including high phosphorus suppressing AMF and sub-deficient calcium limiting nodulation. The value of this combined strategy lies in its ability to simultaneously overcome phosphorus-induced suppression of AMF and calcium-limited nodulation through complementary mechanisms, resulting in enhanced nodulation compared with AMF alone. This promoting effect was further supported by integrated transcriptomic and metabolomic analyses, revealing a multi-level regulatory network in the combined treatment that differs from that of the single treatments. At the metabolic level, CaO specifically activated the PPP pathway, enhancing carbon flux to rhizobia, consistent with the understanding that PPP metabolites serve as a preferred carbon source for rhizobia [46]. Interestingly, the FBP-encoding gene (*AH20G09090*) was significantly up-regulated in the AMF+CaO treatment, yet sucrose content decreased. This apparent contradiction reflects the dynamic nature of carbon partitioning: FBP up-regulation may drive sucrose synthesis, but the rapidly increasing carbon demand for nodule organogenesis, maintenance, and nodule formation and maintenance likely channels sucrose-derived intermediates into glycolysis and PPP to fuel these energy-costly symbiotic processes. At the membrane and transport level, enrichment of unsaturated fatty acid biosynthesis and ABC transporter pathways was observed in the AMF+CaO treatment, indicating enhanced lipid transport and increased membrane fluidity [47]. At the signaling level, the up-regulation of phenylpropanoid and protein export pathways suggests that calcium enhances flavonoid signaling and rhizobium recruitment [48]. These findings indicate that the role of calcium extends beyond traditional nutritional complementarity to function as a systemic metabolic coordinator; when combined with AMF, calcium integrates carbon allocation, membrane transport, and chemical signaling into a unified symbiotic program. This study also has certain limitations. The experiments were conducted under greenhouse pot-grown conditions, and the agronomic applicability of the AMF+CaO promoting effect requires further validation through field trials under different soil types and environmental conditions. Furthermore, although transcriptomic analysis identified key genes and metabolic pathways, their causal roles in mediating the observed effects still need to be functionally validated through genetic approaches such as gene knockout and overexpression. In addition, the current experimental design did not include a CaO-only treatment, which would be necessary to formally distinguish additive from joint effects between AMF and CaO. It should also be noted that soil pH was not measured in this study; therefore, we cannot rule out the possibility that CaO-induced alterations in soil pH may have partially contributed to the observed transcriptomic and metabolomic responses. However, given the relatively low application rate of CaO compared with typical field liming requirements, we consider such pH effects likely to be minor relative to the direct physiological effects of calcium ions. Nevertheless, this aspect warrants further investigation in future studies. Future experiments incorporating CaO alone, AMF alone, AMF+CaO, and a double control will help to formally validate the nature of this relationship. Future research should focus on conducting multi-site field trials to evaluate the robustness of the AMF+CaO promoting effect under actual cultivation conditions. At the same time, emerging peanut genetic engineering technologies should be utilized to functionally characterize key genes, thereby providing a theoretical basis for optimizing combined AMF+CaO application strategies and reducing the use of chemical nitrogen fertilizers in peanut cultivation.

## 4. Material and Methods

### 4.1. Experimental Design

This study was conducted at the greenhouse located within a peanut experimental field of the Shandong Peanut Research Institute (Laixi City, Shandong Province, China, E 120°29′, N 36°48′), which is characterized by a temperate maritime monsoon climate (mean annual temperature 11.3 °C, mean annual precipitation 732 mm). Greenhouse conditions were maintained at 25 ± 3 °C with natural photoperiod and relative humidity of 60%. The peanut variety provided for the experiment was *Arachis hypogaea* L. cv. ‘Huayu 22’ (proprietary to our institute), a widely cultivated genotype with a stable genetic background and high AMF compatibility. Before being sown in soil, all seeds were surface-sterilized with 15% H_2_O_2_ and rinsed with sterile water. The pots had a top diameter of 30.0 cm, a bottom diameter of 24.0 cm, and a height of 26.0 cm, each containing 10.0 kg of soil. The soil was collected from the top layer of 0–20 cm of the peanut production area within the same experimental field. The basic physicochemical properties of the soil were determined according to the methods described in reference [4]. The baseline nutrient status was as follows: available nitrogen 71.06 mg·kg^−1^, available phosphorus 61.06 mg·kg^−1^, available potassium 62.85 mg·kg^−1^, and exchangeable calcium 238 mg·kg^−1^. Analytical grade calcium oxide (CaO, purity ≥ 96.0%) was purchased from Sinopharm Chemical Reagent Co., Ltd. (Shanghai, China) and applied to the soil before sowing. The CaO powder was thoroughly mixed with the entire volume of soil in each pot at a rate of 2.5 g per pot, and no additional CaO was supplied during the subsequent growth period. The AMF inoculum of *Funneliformis mosseae* (T.H. Nicolson & Gerd.) C. Walker & A. Schüßler was kindly provided by Prof. Shaoxia Guo at Qingdao Agricultural University, China. The AMF was propagated on *Zea mays* in a sterilized substrate consisting of river sand, vermiculite, and peat (1:4:1, *v*/*v*), and contained approximately 30 spores·g^−1^. Three treatments were set up in this study: (i) non-inoculated control without CaO (Control), (ii) AMF inoculation alone (AMF), and (iii) combined AMF inoculation and CaO application (AMF+CaO). It should be noted that a CaO-only treatment was not included in the current experimental design; therefore, the individual effect of CaO cannot be formally separated from its combined effect with AMF. Each treatment consisted of 50 pots, and each pot contained 3 peanut plants, ensuring sufficient biological replication for reliable statistical analysis. For the AMF and AMF+CaO treatments, 20 g of this inoculum was placed 5 cm below the soil surface at the time of sowing. Plants in the control treatment received the same amount of sterilized inoculum (autoclaved at 121 °C for 20 min twice) to eliminate any potential effects of the carrier material and its associated non-target microorganisms. Throughout the experiment, pots were irrigated regularly with 1000 mL of tap water every two days to maintain consistent soil moisture. All pots were maintained under identical growth conditions and were regularly rotated to minimize positional effects.

### 4.2. AMF Colonization Assessment

Root samples were cleared with 10% KOH, acidified with 1% HCl, and stained with 0.05% trypan blue in lactoglycerol. Mycorrhizal colonization was evaluated under a light microscope using the gridline intersect method [49]. At least 100 root segments per sample were examined, and colonization rate was calculated as the percentage of root segments containing AMF structures (hyphae, arbuscules, or vesicles).

### 4.3. Root and Nodule Phenotypic Analysis

After being cultured in the greenhouse, 19 pots were randomly selected from the 50 pots per treatment, and one plant was randomly taken from each pot. Plants were separated into shoots (stems, leaves, and flowers) and roots (all below-ground organs excluding pods). All shoot and root samples were oven-dried at 70 °C to constant weight and weighed. For nodule number and nodule weight, 12 of the 19 pots were randomly subsampled; nodules from each selected plant were meticulously dissected from the entire root system and counted individually under a stereomicroscope, then pooled, oven-dried, and weighed.

### 4.4. RNA Extraction and RNA-Sequencing

All samples for transcriptomic analyses were collected at 70 days after sowing. For each biological replicate, nine individual plants from the same treatment were randomly selected (from three randomly chosen pots, with three plants per pot), and their roots were excised, and nodules were carefully removed before the root tissues were pooled and immediately frozen in liquid nitrogen, then stored at −80 °C until further use. A total of three independent biological replicates were prepared for each treatment. Total RNA from each sample was extracted with the RNAprep Pure Plant Kit (Tiangen, Beijing, China) according to the manufacturer’s instructions. RNA purity was assessed by measuring the A260/A280 absorbance ratio using a Nanodrop ND-2000 spectrophotometer (Thermo Scientific, Waltham, MA, USA), and RNA integrity was evaluated using an Agilent Bioanalyzer 4150 (Agilent Technologies, Santa Clara, CA, USA) to determine the RNA integrity number (RIN). Only RNA samples with a qualified quality (A260/A280 ratio between 1.8 and 2.0, and RIN ≥ 7.0) were used for subsequent library construction. After the mRNA was tested for purity and concentration, it was enriched by using magnetic beads containing Oligo (dT) and then randomly disrupted with fragmentation buffer. Using random hexamers as templates, the first strand of cDNA was synthesized. Buffer, dNTPs, and DNA polymerase I were then added to synthesize the second strand. The double-stranded cDNA was then purified using AMPure XP beads (Beckman Coulter, Inc., Brea, CA, USA), followed by end repair, A-tail addition, and ligation to a sequencing junction, and then fragment size selection was performed with AMPure XP beads, and finally PCR enrichment was performed to obtain the final cDNA library. The final constructed cDNA library was sequenced on an Illumina Novaseq 6000 platform at Applied Protein Technology Co., Shanghai, China. Each sample yielded approximately 6 Gb of clean data with 150 bp paired-end reads, providing sufficient sequencing depth for transcriptome analysis.

Raw sequencing data in FASTQ format were processed using Fastp (v0.23.2) for quality control. Adapter sequences were trimmed, and low-quality reads were filtered out using the following criteria: reads with more than 60% of bases having a quality score ≤ 25, or with more than 5% ambiguous bases (N), were discarded. The resulting clean reads were retained for subsequent analyses. The clean reads were aligned to the reference cultivated peanut genome assembly (accession: PRJNA480120) using Hisat2 (v2.2.1). Gene annotation was based on the corresponding GFF3 annotation file from the same genome version. Both the genome assembly and annotation data are publicly accessible at http://peanutgr.fau.edu.cn/Download.php (accessed on 15 October 2025) [50]. The overall mapping rate for all samples exceeded 85%. PCA biplot was generated using the R stats package prcomp function. Gene abundance in peanut roots was assessed through FPKM normalization (Fragments Per Kilobase of transcript per Million fragments mapped). FeatureCounts (http://subread.sourceforge.net/) (accessed on 20 October 2025)was used to count the read numbers mapped to each gene. And then FPKM of each gene was calculated based on the length of the gene and reads count mapped to this gene. All genes were screened with |log2(fold change)| ≥ 1 and adjusted *p*-value < 0.01 to obtain the differentially expressed genes (DEGs) by using the DESeq2 R package (v1.34.0). The metabolic pathways involved in the DEGs were further analyzed with reference to the KEGG (Kyoto Encyclopedia of Genes and Genomes) database. Gene Ontology (GO) annotation was performed using the Blast2GO (v2.5.0), and Kyoto Encyclopedia of Genes and Genomes (KEGG) orthology assignment was conducted using kobas-annotate (v3.0.3). GO and KEGG enrichment analyses were carried out using the clusterProfiler R package (v4.6.2), with a significance threshold of adjusted *p*-value < 0.05.

### 4.5. Metabolites’ Extraction and Analysis

All samples for metabolomic analyses were collected at the same time as those for transcriptomic analysis. Six biological replicates were prepared per treatment: three derived from the same pooled root tissues used for transcriptomics (enabling direct Pearson correlation analysis), and three additional replicates randomly selected from additional pots within the same treatment. For each biological replicate, nine plants were randomly selected from the same treatment group (from three randomly chosen pots, with three plants per pot). Eighty milligrams of root tissue of each sample were quickly frozen in liquid nitrogen immediately and grounded into fine powder with a mortar and pestle. A total of 1000 μL methanol/acetonitrile /H_2_O (2:2:1, *v*/*v*/*v*) were added to homogenized solution for metabolite extraction. The mixture was centrifuged for 20 min (14,000× *g*, 4 °C). The supernatant was dried in a vacuum centrifuge. For LC-MS analysis, samples were re-dissolved in 100 μL of acetonitrile/water (1:1, *v*/*v*) solvent and centrifuged at 14,000× *g* at 4 °C for 15 min, after which the supernatant was injected. Analyses were performed using an UHPLC (1290 Infinity LC, Agilent Technologies, Santa Clara, CA, USA) coupled to a quadrupole time-of-flight (AB Sciex TripleTOF 6600, AB Sciex, Framingham, MA, USA) at Shanghai Applied Protein Technology Co., Ltd. (Shanghai, China).

The raw MS data were converted to MzXML files using ProteoWizard MSConvert (v3.0.23078) before importing into freely available XCMS software (v4.6.4). Metabolite identification was achieved by matching experimental measurements against an in-house reference database (Shanghai Applied Protein Technology) containing authenticated standards, with acceptance criteria set at mass accuracy (<10 ppm) and MS/MS spectral similarity [51,52]. After normalized to total peak intensity, the processed data were analyzed by ropls R package (v1.28.0) package, where it was subjected to multivariate data analysis, including Pareto-scaled principal component analysis (PCA) and orthogonal partial least-squares discriminant analysis (OPLS-DA). Model reliability was assessed through 7-fold cross-validation and response permutation testing. The variable importance in the projection (VIP) value of each variable in the OPLS-DA model was calculated to indicate its contribution to the classification. Metabolites with the VIP value >1 was further applied to Student’s *t*-test at univariate level to measure the significance of each metabolite, the *p*-value < 0.05 was considered as statistically significant. The significant difference accumulated metabolites (DAMs) associated metabolic pathways were further analyzed with reference to the KEGG (Kyoto Encyclopedia of Genes and Genomes) database. KEGG enrichment analyses were carried out using the clusterProfiler R package (4.6.2), with a significance threshold of adjusted *p*-value < 0.05. The pathway impact plot was generated using MetaboAnalyst (https://www.metaboanalyst.ca) (accessed on 10 December 2025). The volcano plots, heatmaps and chord diagrams illustrating differential metabolites under different treatments were constructed using the APT-BioCloud online platform (APTBIO, Shanghai, China). To visualize co-regulation relationships among metabolite classes, chord diagrams was constructed based on the correlation matrix of all metabolites. Pearson correlation coefficients were calculated for every pairwise combination of metabolites using their relative abundance across all biological replicates. Only metabolite pairs with |r| > 0.8 and *p* < 0.05 were retained for further analysis. Each metabolite was then assigned to a chemical class according to the HMDB classification. For each pair of metabolite classes, we counted the number of significantly correlated metabolite pairs connecting the two classes; this count was defined as the transition weight between the two classes. In the chord diagram, the width of the chord connecting two class sectors is proportional to this weight, with wider chords indicating a greater number of significant inter-class correlations and thus stronger co-regulation between the two classes. Chord widths were scaled relative to the maximum weight within each comparison group to ensure comparability across treatments. The underlying transition weight matrices are provided in Table S5.

### 4.6. Quantitative Real-Time PCR

Total RNA from each sample was extracted with the RNAprep Pure Plant Kit (Tiangen, Beijing, China) according the instructions of manufacturer. After measuring the RNA purity and concentration, total RNA was transcribed into cDNA using the FastKing gDNA Dispelling RT SuperMix kit (Takara Bio Inc., Shiga, Japan) (RT). For quantitative analysis, we conducted real-time PCR (qRT-PCR) on an ABI Step One Plus platform (Applied Biosystems, Carlsbad, CA, USA) with TB Green™ Premix Ex Taq™ II (Tli RNase H Plus) (TaKaRa, Japan), employing the following thermal profile: initial denaturation at 95 °C for 10 min, followed by 40 cycles of 95 °C for 15 s and 60 °C for 60 s. Gene expression levels were normalized to *Ahactin11* as an internal control and quantified using the 2^−ΔΔCT^ method. Nine genes were randomly selected for verifying the accuracy of high-throughput sequencing results. Primers used for qRT-PCR were given in Table S7.

### 4.7. Statistical Analysis

Statistical significance was determined by one-way ANOVA followed by Tukey’ s post hoc test for multiple comparisons. Bar charts with significance letters were generated using the Chiplot online platform (https://www.chiplot.online/) (accessed on 20 December 2025). For Pearson correlation analysis between DEGs and DAMs, we used only the three matched biological replicates derived from the same pooled root tissues. The additional three metabolomics replicates were excluded from this paired analysis to ensure valid correlation estimates. Pearson correlation analysis was performed using R (v4.3.2) to calculate correlation coefficients and *p*-values for each gene-metabolite pair. The resulting correlation matrix was visualized as a heatmap using the Hiplot platform (https://hiplot.com.cn).

## 5. Conclusions

**Figure 8:**
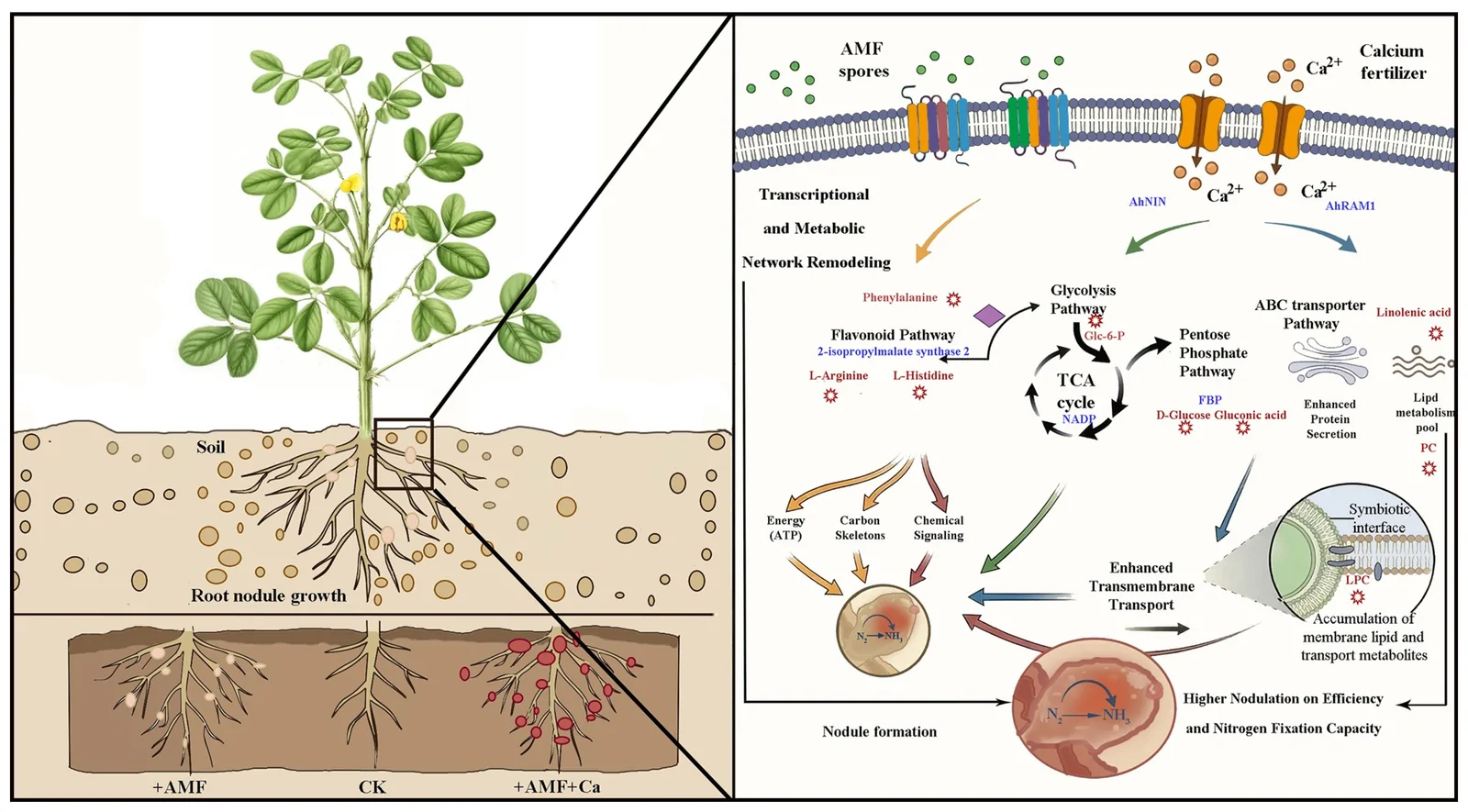
Proposed mechanistic model of AMF and calcium fertilizer jointly promoting peanut nodulation. Red stars represent representative DAMs in each pathway, and blue words indicate DEGs mapped to the corresponding pathways. Black solid arrows represent biological processes leading to developmental and functional symbiotic phenotypic outcomes. Colored directional arrows indicate conceptual regulatory relationships between distinct biological modules, with colors used solely for visual discrimination and no specific functional implication. *AH19G28260*: NADP; *AH20G09090*: FBP; *AH12G21400*: 2-isopropylmalate synthase. Lyso-phosphatidylcholine: LPC; Phosphatidylcholine: PC.

The combined application of AMF and calcium fertilizer in promoting peanut nodulation involves multiple interconnected processes. AMF colonization was associated with altered carbon and nitrogen metabolic profiles and transcriptional changes in phenylpropanoid metabolism-related pathways, which may be relevant to energy metabolism, carbon skeleton availability, and signaling molecule production for nodule formation. The addition of calcium to AMF inoculation was associated with additional transcriptomic and metabolomic changes involving the glyoxylate cycle and pentose phosphate pathway, which may contribute to energy and reducing power associated with nodule development. Additionally, calcium was observed to correlate with expression changes in genes related to protein secretion and lipid metabolism, as well as altered abundance of metabolites associated with membrane lipids and transport. These changes co-occurred with an increased number of nodules per peanut plant (Figure 8).

This study details the multi-level mechanisms by which AMF combined with calcium fertilizer correlates with enhanced peanut nodulation from a systems metabolism perspective, providing a descriptive framework for the complex relationships among plants, microbes, and calcium. While the observed promoting effect of combined AMF and calcium application is evident, formal distinction between additive and synergistic contributions will require future experimental designs that include a CaO-only treatment. Future research could focus on screening or engineering AMF strains with superior symbiotic efficiency and optimizing the timing and dosage of calcium fertilizer application. Such approaches may be useful for reducing chemical nitrogen fertilizer inputs while possibly improving the biological nitrogen fixation potential and field adaptability of leguminous crops like peanuts, thus supporting the development of resource-efficient and environmentally friendly modern agricultural systems.

## Data Availability

The transcriptomic and metabolomic data that support the findings of this study have been deposited into CNCB (China National Center for Bioinformation) with accession number PRJCA054444 and OMIX019565.

## Supplementary Materials

The following supporting information can be downloaded at: https://www.mdpi.com/article/10.3390/plants15172640/s1, Figure S1. PCA score plot of transcriptome data from peanut roots subjected to Control, AMF, and AMF+CaO treatments. Each point represents a biological replicate (*n* = 3). Figure S2. Validation of transcriptome data by quantitative real-time PCR. Relative expression levels of selected genes were consistent with RNA-seq results, and different lowercase letters indicate statistically significant differences among treatments (*p* < 0.05). Figure S3. PCA score plots of metabolic profiles in negative (NEG) and positive (POS) ionization modes for Control, AMF, and AMF+CaO groups. Each point represents a biological replicate (*n* = 6). Table S1. AMF colonization rates in peanut roots under different treatments. Values are mean ± SD (*n* = 6 per treatment). Table S2. DEGs in comparison of AMF-v-Control, AMF+CaO-v-Control and AMF+CaO-v-AMF. Table S3. DEGs of AMF-v-Control, AMF+CaO-v-Control, AMF+CaO-v-AMF enriched metabolic pathways. Table S4. DAMs in comparison of AMF-v-Control, AMF+CaO-v-Control and AMF+CaO-v-AMF. Table S5. Weight matrix of significant correlations between metabolite classes. Table S6. Correlation matrix of DEGs and DAMs in the transcriptome–metabolome association analysis. Table S7. Primers used for qRT-PCR.

## References

[1] U.S. Department of Agriculture (2025). World Agricultural Supply and Demand Estimates (WASDE-672). https://www.usda.gov/.

[2] Mitra M., Jainab S.I.B., David K.M., Sunitha S., Bhandari A., Nagaraju D., Hadimani L.G. (2025). Role of Rhizobium and non-symbiotic plant growth promoting rhizobacteria in nitrogen fixation and growth of *Arachis hypogea*: A review. Int. J. Plant Soil Sci..

[3] Lu Y., Dai H., Zamanian K., Sun Q., Wang C., Wu Z., Zheng Y., Li L., Wan S., Yu T. (2023). Liming shifts the chemical properties and microbial community structures of peanut soils with different initial pH values. Appl. Soil Ecol..

[4] Wang J., Geng Y., Zhang J., Li L., Guo F., Yang S., Zou J., Wan S. (2023). Increasing Calcium and Decreasing Nitrogen Fertilizers Improves Peanut Growth and Productivity by Enhancing Photosynthetic Efficiency and Nutrient Accumulation in Acidic Red Soil. Agronomy.

[5] Smith S.E., Read D.J. (2010). Mycorrhizal Symbiosis.

[6] Lodwig E.M., Hosie A.H.F., Bourdès A., Findlay K., Allaway D., Karunakaran R., Downie J.A., Poole P.S. (2003). Amino-acid cycling drives nitrogen fixation in the legume–Rhizobium symbiosis. Nature.

[7] Scheublin T.R., Van Der Heijden M.G. (2006). Arbuscular mycorrhizal fungi colonize nonfixing root nodules of several legume species. New Phytol..

[8] Wang X.L., Feng H., Wang Y.Y., Wang M.X., Xie X.G., Chang H.Z., Wang L., Qu J.C., Sun K., He W. (2021). Mycorrhizal symbiosis modulates the rhizosphere microbiota to promote rhizobia–legume symbiosis. Mol. Plant.

[9] Shi J.C., Zhao B.Z., Zheng S., Zhang X.W., Wang X.L., Dong W.T., Xie Q.J., Wang G., Xiao Y.P., Chen F. (2021). A phosphate starvation response-centered network regulates mycorrhizal symbiosis. Cell.

[10] Ding X., Zhang S., Wang R., Li S., Liao X. (2016). AM fungi and rhizobium regulate nodule growth, phosphorous (P) uptake, and soluble sugar concentration of soybeans experiencing P deficiency. J. Plant Nutr..

[11] Kudriashova T.R., Kryukov A.A., Gorbunova A.O., Gorenkova A.I., Kovalchuk A.I., Shishova M.F., Yurkov A.P. (2024). The effect of arbuscular mycorrhiza on gene expression of sweet transporters in *Medicago lupulina* under conditions of high available phosphorus level. Ecol. Genet..

[12] Lanfranco L., Fiorilli V., Venice F., Bonfante P. (2018). Strigolactones cross the kingdoms: Plants, fungi, and bacteria in the arbuscular mycorrhizal symbiosis. J. Exp. Bot..

[13] Banasiak J., Borghi L., Stec N., Martinoia E., Jasiński M. (2020). The full-size ABCG transporter of *Medicago truncatula* is involved in strigolactone secretion, affecting arbuscular mycorrhiza. Front. Plant Sci..

[14] Qin W., Li G., Chen X., Liu J. (2024). Organic amendments enhance peanut nodulation by influencing interactions between rhizobia and arbuscular mycorrhizal fungi in the peanut rhizosphere. Agronomy.

[15] Bi Y., Zhou H. (2021). Changes in peanut canopy structure and photosynthetic characteristics induced by an arbuscular mycorrhizal fungus in a nutrient-poor environment. Sci. Rep..

[16] Feng D., Wang X., Gao J., Zhang C., Liu H., Liu P., Sun X. (2023). Exogenous calcium: Its mechanisms and research advances involved in plant stress tolerance. Front. Plant Sci..

[17] Yan L., Liu S., Li R., Li Z., Piao J., Zhou R. (2024). Calcium enhanced the resistance against Phoma arachidicola by improving cell membrane stability and regulating reactive oxygen species metabolism in peanut. BMC Plant Biol..

[18] Weng X., Li H., Ren C., Zhou Y., Zhu W., Zhang S., Liu L. (2022). Calcium regulates growth and nutrient absorption in poplar seedlings. Front. Plant Sci..

[19] Luan L., Wang C. (2021). Calcium signaling mechanisms across kingdoms. Annu. Rev. Cell Dev. Biol..

[20] Capoen W., Sun J., Wysham D., Otegui M.S., Venkateshwaran M. (2011). Nuclear membranes control symbiotic calcium signaling of legumes. Proc. Natl. Acad. Sci. USA.

[21] Ke D., Fang Q., Chen C., Zhu H., Chen T., Chang X., Yuan S., Kang H., Ma L., Hong Z. (2012). The small GTPase ROP6 interacts with NFR5 and is involved in nodule formation in Lotus japonicus. Plant Physiol..

[22] Ma Y., Xiao C., Liu J., Ren G. (2025). Nutrient-dependent regulation of symbiotic nitrogen fixation in legumes. Hortic. Res..

[23] Krylova V.V., Andreev I.M., Dubrovo P.N., Andreeva I.N., Izmailov S.F. (2004). Calcium in the control of nitrogenase activity in Vicia faba L. root nodules: Calcium release from symbiosomes and the accompanying decrease in their nitrogenase activity is blocked by verapamil. Biol. Bull..

[24] Kim W., Acosta-Jurado S., Kim S., Krishnan H.B. (2024). Calcium induces the cleavage of NopA and regulates the expression of nodulation genes and secretion of T3SS effectors in Sinorhizobium fredii NGR234. Int. J. Mol. Sci..

[25] Li C., Feng G., Yang Y., Meng J., Geng Y., Wang Q., Wang S. (2019). Arbuscular mycorrhizal fungi combined with exogenous calcium improves the growth of peanut (*Arachis hypogaea* L.) seedlings under continuous crop. J. Integr. Agric..

[26] Bhattacharjee O., Raul B., Ghosh A., Bhardwaj A., Bandyopadhyay K., Sinharoy S. (2022). Nodule INception-independent epidermal events lead to bacterial entry during nodule development in peanut (*Arachis hypogaea*). New Phytol..

[27] Gao J.P., Kumar A. (2025). RAM1-WRI Synergy: A GRAS-AP2 Regulatory Axis for Nutrient Exchange in Arbuscular Mycorrhizal Symbiosis. Plant Cell Environ..

[28] Lindström K., Mousavi S.A. (2020). Effectiveness of nitrogen fixation in rhizobia. Microb. Biotechnol..

[29] He J., Zhang L., Van Dingenen J., Desmet S., Goormachtig S., Calonne-Salmon M., Declerck S. (2024). Arbuscular mycorrhizal hyphae facilitate rhizobia dispersal and nodulation in legumes. ISME J..

[30] Granqvist E., Sun J., den Camp R.O., Pujic P., Hill L., Normand P., Morris R.J., Downie J.A., Geurts R., Oldroyd G.E.D. (2015). Bacterial-induced calcium oscillations are common to nitrogen-fixing associations of nodulating legumes and nonlegumes. New Phytol..

[31] Zhang Q., Blaylock L.A., Harrison M.J. (2010). Two *Medicago truncatula* half-ABC transporters are essential for arbuscule development in arbuscular mycorrhizal symbiosis. Plant Cell.

[32] Bravo A., Brands M., Wewer V., Dörmann P., Harrison M.J. (2017). Arbuscular mycorrhiza-specific enzymes FatM and RAM 2 fine-tune lipid biosynthesis to promote development of arbuscular mycorrhiza. New Phytol..

[33] Sin W.C., Liu J., Zhong J.Y., Lam H.M., Lim B.L. (2024). Comparative proteomics analysis of root and nodule mitochondria of soybean. Plant Cell Environ..

[34] Li Y., Lu L., Wang Q., Liu X., Tian J., Zhang R., Liao H., Lambers H., Wang X. (2025). Arbuscular mycorrhizal fungi promote nodulation and N_2_ fixation in soybean by specific root exudates. Plant Cell Environ..

[35] Hassan S., Mathesius U. (2012). The role of flavonoids in root-rhizosphere signaling: Opportunities and challenges for improving plant-microbe interaction. J. Exp. Bot..

[36] Poole P., Ramachandran V., Terpolilli J. (2018). Rhizobia: From saprophytes to endosymbionts. Nat. Rev. Microbiol..

[37] Wang D., Griffitts J., Starker C., Fedorova E., Limpens E., Ivanov S., Bisseling T., Long S. (2010). A nodule-specific protein secretory pathway required for nitrogen-fixing symbiosis. Science.

[38] Chinnasamy G., Davis P.J., Bal A.K. (2003). Seasonal changes in oleosomic lipids and fatty acids of perennial root nodules of beach pea. J. Plant Physiol..

[39] Koch M., Delmotte N., Rehrauer H., Vorholt J.A., Pessi G., Hennecke H. (2010). Rhizobial adaptation to hosts, a new facet in the legume root-nodule symbiosis. Mol. Plant-Microbe Interact..

[40] Roy S., Breakspear A., Cousins D., Torres-Jerez I., Jackson K., Kumar A., Su Y., Liu C.W., Krom N., Udvardi M. (2021). Three common symbiotic ABC subfamily B transporters in *Medicago truncatula* are regulated by a NIN-independent branch of the symbiosis signaling pathway. Mol. Plant-Microbe Interact..

[41] Jarzyniak K., Banasiak J., Jamruszka T., Pawela A., Di Donato M., Novák O., Geisler M., Jasiński M. (2021). Early stages of legume-rhizobia symbiosis are controlled by ABCG-mediated transport of active cytokinins. Nat. Plants.

[42] Bournaud C., James E.K., de Faria S.M., Lebrun M., Melkonian R., Duponnois R., Tisseyre P., Moulin L., Prin Y. (2018). Interdependency of efficient nodulation and arbuscular mycorrhization in Piptadenia gonoacantha, a Brazilian legume tree. Plant Cell Environ..

[43] Larimer A.L., Clay K., Bever J.D. (2014). Synergism and context dependency of interactions between arbuscular mycorrhizal fungi and rhizobia with a prairie legume. Ecology.

[44] Gleason C., Chaudhuri S., Yang T., Muñoz A., Poovaiah B.W., Oldroyd G.E. (2006). Nodulation independent of rhizobia induced by a calcium-activated kinase lacking autoinhibition. Nature.

[45] Diao R.N., Yang S., Zhang J.L., Wang J., Peng Z., Yu X., Li X., Wan S. (2022). The mechanisms of calcium regulation on peanut nodulation and nitrogen fixation analyzed by transcriptomes and metabonomics. J. Peanut Sci..

[46] Mellor R.B., Werner D. (2018). Legume nodule biochemistry and function. Molecular Biology of Symbiotic Nitrogen Fixation.

[47] Lu B.F., Kang W.J., Shi S.L., Jing F., Guan J. (2025). Comparative transcriptomics reveals differential carbohydrate and lipid metabolism in roots and nodules of Rhizobia-Inoculated alfalfa (*Medicago sativa* L.). BMC Plant Biol..

[48] Cui L., Guo F., Zhang J., Yang S., Meng J., Geng Y., Li X., Wan S. (2020). Synergy of arbuscular mycorrhizal symbiosis and exogenous Ca benefits peanut (*Arachis hypogaea* L.) growth through the shared hormone and flavonoid pathway. Sci. Rep..

[49] Phillips J.M., Hayman D.S. (1970). Improved procedures for clearing roots and staining parasitic and vesicular-arbuscular mycorrhizal fungi for rapid assessment of infection. Trans. Br. Mycol. Soc..

[50] Zhuang W., Chen H., Yang M., Wang J., Pandey M.K., Zhang C., Nipper W.C., Zhang X. (2019). The genome of cultivated peanut provides insight into legume karyotypes, polyploid evolution and crop domestication. Nat. Genet..

[51] Luo D., Deng T., Yuan W., Deng H., Jin M. (2017). Plasma metabolomic study in Chinese patients with wet age-related macular degeneration. BMC Ophthalmol..

[52] Gu Z., Li L., Tang S., Liu C., Fu X., Shi Z., Mao H. (2018). Metabolomics reveals that crossbred dairy buffaloes are more thermotolerant than Holstein cows under chronic heat stress. J. Agric. Food Chem..

